# Past, present, and future of cartilage restoration: from localized defect to arthritis

**DOI:** 10.1186/s43019-022-00132-8

**Published:** 2022-01-28

**Authors:** Dong Hwan Lee, Seok Jung Kim, Seon Ae Kim, Gang-ik Ju

**Affiliations:** Department of Orthopedic Surgery, Uijeongbu St. Mary’s Hospital, College of Medicine, The Catholic University of Korea, 271, Cheonbo-ro, Gyeonggi-do 11765 Uijeongbu-si, Republic of Korea

**Keywords:** Minimally invasive surgery, Osteoarthritis, Arthritis, Biological treatment

## Abstract

**Background:**

Osteoarthritis, one of the most common joint diseases, is characterized by the loss of joint function due to articular cartilage destruction. Herein, we review current and previous research involving the clinical applications of arthritis therapy and suggest potential therapeutic options for osteoarthritis in the future.

**Past, present, and future treatment:**

The arthroscopic cartilage regeneration procedure or realignment osteotomy has been performed as a joint-conserving procedure in cases where conservative treatment for damaged articular cartilage and early osteoarthritis failed. If cartilage regeneration is ineffective or if the joint damage progresses, arthroplasty is the main treatment option. The need for biological arthritis treatment has expanded as the healthy lifespan of the global population has increased. Accordingly, minimally invasive surgical treatment has been developed for the treatment of damaged cartilage and early osteoarthritis. However, patients generally prefer to avoid all types of surgery, including minimally invasive surgery. Therefore, in the future, the treatment of osteoarthritis will likely involve injection or medication.

**Conclusion:**

Currently, arthritis management primarily involves the surgical application of therapeutic agents to the joints. However, nonsurgical or prophylactic methods are expected to become mainstream arthritis therapies in the future.

## Background

Although mechanistic details regarding outcomes associated with articular cartilage damage remain unclear, the idea that arthritis occurs in areas surrounding articular cartilage damage is generally accepted [1]. Osteoarthritis, one of the most common joint diseases, occurs in about 70% of individuals aged ≥ 65 years, and is characterized by the loss of joint function due to articular cartilage destruction [2]. Articular cartilage damage has the potential to cause severe pain, which limits an individual’s ability to perform physical activities and, therefore, contributes to additional medical problems [3]. Pain management using medication and physical treatment have long been therapeutic options for the treatment of damaged articular cartilage and early arthritis. In cases where symptoms do not improve, an arthroscopic cartilage regeneration procedure is performed. If cartilage regeneration is ineffective or joint damage progresses, surgical treatment, such as arthroplasty, is the primary treatment option available [4].

As life expectancy increases, patients have tended to have an increasingly strong desire to live a healthy life and maintain their native joints into old age. Therefore, efforts to protect articular cartilage are continuously made [5, 6]. Currently, joint-conserving treatments such as stem cell surgery are commonly used to repair damaged joint cartilage and arthritis. Recently, efforts have been made to preserve joints using nonsurgical methods [7]. Herein, we intend to review past and present research that has examined the clinical applications of arthritis therapy to suggest possible therapeutic options for the future.

## Past

In 1934, Burman was the first to release a report on arthroscopic treatment using the word “arthroscopy” in the title [8]. His findings revealed that patients with arthritis experienced symptom improvement attributable to the removal of mechanical irritants via lavage. The procedure was used because arthroscopy was limited to the examination of joints, given characteristics of surgical instruments and techniques used at that time.

**Fig. 1:**
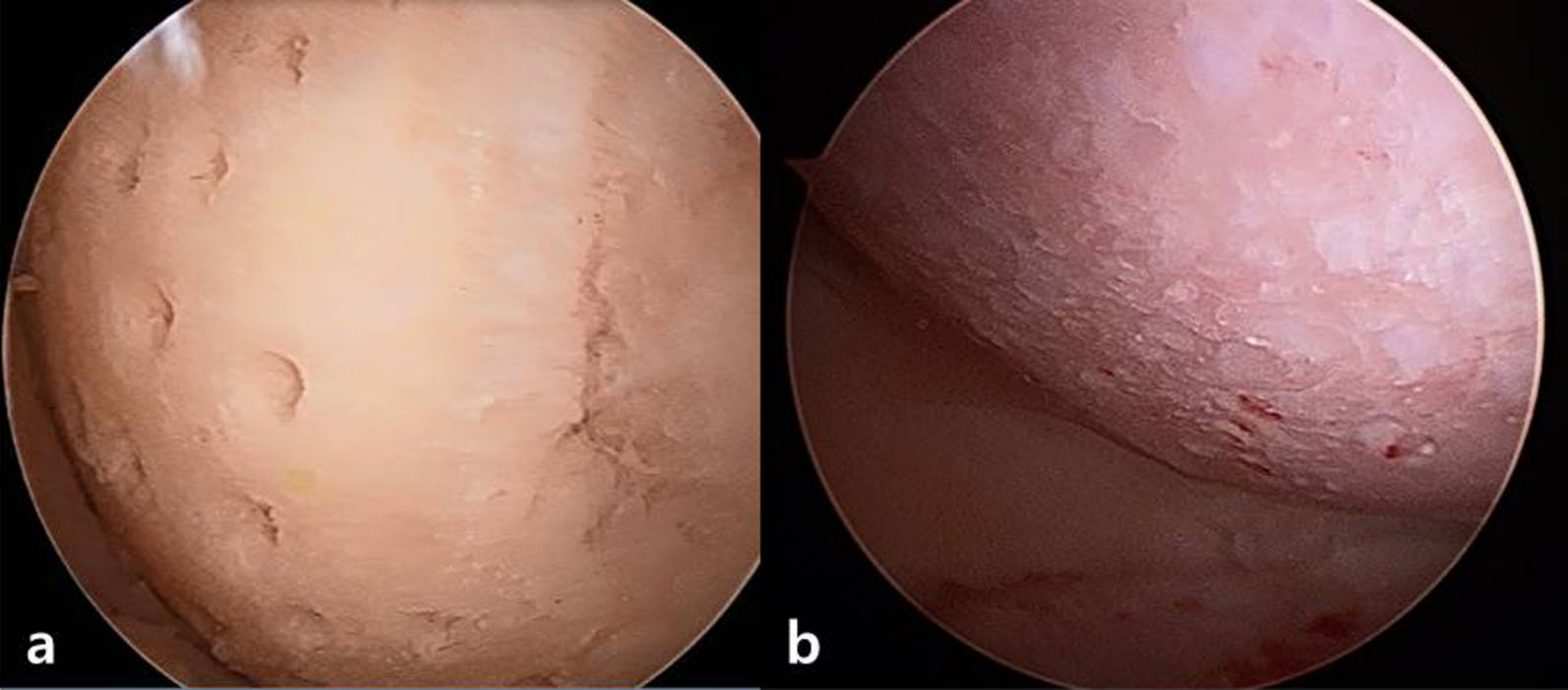
Images of cartilage defects with multiple sites of drilling **a** at the time of surgery, and **b** via arthroscopy 2-years postsurgery are shown

About two decades later, in 1959, Pridie introduced the principle of drilling exposed subchondral bone to treat damaged cartilage [9]. The objective of the procedure was to form fibrocartilage by allowing bone marrow to flow into the area of drilling. This treatment was widely used in Europe, but it was not very effective for relieving symptoms of osteoarthritis (Fig. 1). Later, Rodrigo et al. described the use of arthroscopic microfracture. The procedure required that a sharp awl be used to avoid heat-induced osteonecrosis during drilling [10]. Microfracture was used to treat articular cartilage defects rather than arthritis. Steadman [11] said that using an awl rather than a drill makes the joint surface lesion rough. The technique takes advantage of the fact that the presence of a rough surface facilitates the adherence of blood clots and promotes cartilage regeneration. Since the 1990s, this technique has been considered the primary method for treating cartilage damage [12, 13].

Multiple reports have shown that microfracture may be used to effectively treat local cartilage defects in young patients. Recently, it has become a basic surgical technique in the treatment of local cartilage defects [14, 15]. However, after microfracture surgery, the structure of subchondral bones can weaken. This allows for the formation of a bone cyst within the bone, or a bony spur within the cartilage lesion, which ultimately results in the worsening of symptoms. As such, several basic disadvantages of microfracture have been reported [16, 17].

**Fig. 2:**
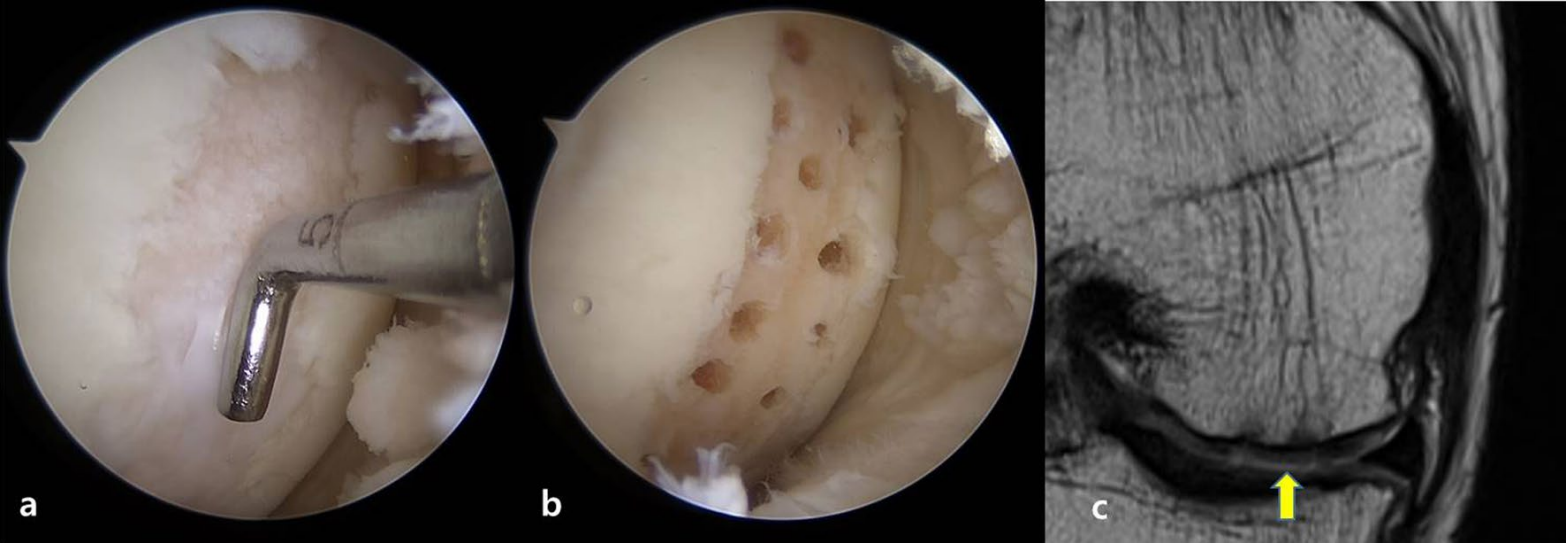
Images of a cartilage defect of the medial femoral condyle **a**, **b** after microfracture was performed, and **c** at 1-year follow-up via magnetic resonance imaging are shown. The yellow arrow indicates repaired tissue

Microfracture treatment involves the creation of a channel in the subchondral bone to allow bone marrow components, including bone marrow mesenchymal stem cells, to flow outward. This allows stem cells to differentiate into chondrocytes and facilitates the formation of cartilage. However, the type of cartilage formed is mostly fibrocartilage, rather than hyaline cartilage, which is the normal articular cartilage type [18]. Fibrocartilage has a high type 1 collagen and low proteoglycan content, and thus is less resistant to wear [19]. Therefore, when fibrocartilage is formed to replace damaged cartilage tissues, 60–70% symptom improvement is expected for approximately 2 years postsurgery. Thereafter, symptom worsening can occur due to structural disintegration [20, 21]. However, several studies have reported good clinical results after microfracture treatment, which were maintained even at a long-term follow-up of more than 2 years; thus, further research is required to comprehensively evaluate the effects of this treatment. [12, 22]. It is known that symptom severity tends to increase as the defect area increases. Therefore, for those with cartilage defects larger than 2 cm^3^, good clinical results are not expected. In fact, the current acceptable cartilage lesion size limit for microfracture is 2 cm^3^ [21, 23]. However, doctors often perform microfracture on patients with larger cartilage lesions due to economic concerns and the simple nature of the surgical procedure. As such, microfracture has emerged as an initial treatment for femoral cartilage damage in the knee joint (Fig. 2).

Lanny Johnson introduced abrasion arthroplasty at the American Academy of Orthopedic Surgeons (AAOS) in 1982, where he reported that two thirds of 103 patients who underwent the therapy obtained satisfactory results [16]. The outcomes of abrasion arthroplasty were better than those of other arthritis treatments, and Dr. Clement, who attended the AAOS conference, said that only Dr. Johnson could achieve such a good result. In 1986, Lanny Johnson reported the use of an arthroscopic surgical technique that involved the removal of 1–3 mm of sclerotic subchondral bone using a burr to expose viable bone and vasculature [24]. Thereafter, several good outcomes of arthroscopic abrasion arthroplasty were reported, and the technique received attention until other studies revealed that abrasion arthroplasty was no better than simple debridement and resulted in severe symptom worsening. In the early 1990s, arthroscopic abrasion arthroplasty was only rarely used [25, 26] Later, Dandy reported that the technique is suitable for small cartilage defects, a drilling technique should be used for large cartilage defects to maintain the structure of subchondral bone [27]. Few additional reports on the topic were available until a recent long-term follow-up study was published. Findings of the study revealed success rates similar to those that were reported initially by Lanny Johnson. Therefore, some researchers continue to consider abrasion arthroplasty a reliable technique [28, 29].

In the past, joint problems in patients with arthritis were mainly treated because there were limited strategies for objectively measuring and diagnosing articular cartilage damage. In fact, the methods often depended on plain radiographs. However, in the 1990s, magnetic resonance imaging (MRI) emerged as a tool for diagnosing joint problems. Studies revealed that MRI could be used to identify articular cartilage damage as the cause of joint problems [30].

## Present

The most important changes that facilitated the shift from past to present treatment strategies were the efforts made to overcome limitations due to the histological characteristics of articular cartilage [18]. The density of chondrocytes in articular cartilage is low, and it contains a comparatively larger portion of extracellular matrix (ECM). Articular cartilage has a low capacity to regenerate after damage than other tissues due the limited ability of chondrocytes to migrate to areas of articular cartilage damage [31–33]. Consequently, methods that aim to increase the number of chondrocytes or enhance chondrocyte migration or aggregation will be needed to overcome the unfavorable histological characteristics of articular cartilage.

After autologous chondrocyte implantation (ACI) was first performed by Brittberg [34] in 1994, cartilage treatment using cells was developed rapidly. To perform the procedure, a small amount (200–300 mg) of articular cartilage is collected from a minor load-bearing area, and chondrocytes are subsequently isolated and cultured for future implantation. Progress has been made regarding the use of this technique. Currently, ACI is categorized into different generations based on cell culture technique and surgical method.

First-generation ACI was developed and used by Brittberg [35]. In first-generation ACI, a cartilage defect is covered with a periosteal flap taken from the proximal medial tibia. Then, chondrocytes are cultured and subsequently injected. Several studies have reported that first-generation ACI may be performed with good results. However, the technique is technically difficult since it involves the collection of the periosteal flap and tight suturing to prevent cultured chondrocyte leakage. Further, issues may occur due to a large incision or the dedifferentiation and calcification of cultured chondrocytes [35–38].

Second-generation ACI involves the seeding of chondrocytes in a bioscaffold, such as a collagen membrane or fibrin glue, for culture and mixing. They are then implanted within the defect area [39, 40]. These methods do not require an additional incision for collecting the periosteal flap, and they instead allow surgeons to use either a small incision or arthroscopic surgery alone. Therefore, their use is advantageous due to rapid postsurgical rehabilitation and recovery [36, 40].

Third-generation ACI is performed by implanting cell pellets within the area of the cartilage defect via a specialized culture technique that does not involve use of a bioscaffold. This technique increases the capacity of cartilage to regenerate [41–44]. ACI is mainly applied in the treatment for damaged cartilage, and is not generally recommended for treating arthritis.

Human umbilical cord blood‐derived mesenchymal stem cells (hUCB-MSC) have been approved for use with medical products. Various clinical findings associated with hUCB-MSC use have been reported [45, 46]. Cord blood remains in the placenta and umbilical cord after childbirth and has been mainly used for bone marrow transplantation because it contains ≥ tenfold the hematopoietic stem cells than those in the bone marrow of an adult. After it was revealed that a large number of mesenchymal stem cells can be found in the Whartone’s jelly of an umbilical cord, the use of cord blood in cell therapy increased [47]. Although the differentiation capacity of the stem cells of the umbilical cord is limited compared with that of embryonic stem cells, it is greater than that of adult stem cells. Furthermore, the collection process is safe, and is associated with no ethical and tumorigenic problems, as are encountered when using embryonic stem cells [48]. Umbilical cord stem cell use is also associated with a decreased rate of immune rejection compared with other cells [49]. Since there is a great probability that they may be used for allogenic stem cell therapy, commercial products containing umbilical cord stem cells have been developed [50, 51].

**Fig. 3:**
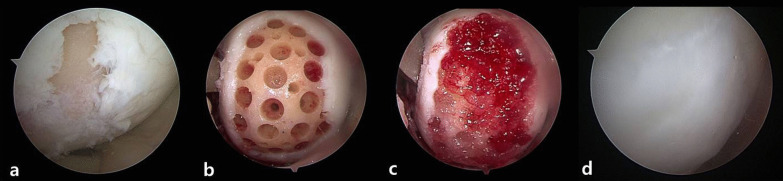
Images of a cartilage defect of medial femoral condyle are shown **a**, **b** after undergoing drilling at multiple sites, **c** when CARTISTEM® was applied to the defect, and **d** via second-look arthroscopy performed 2-years postsurgery

CARTISTEM is an hUCB-MSC product approved for the treatment of osteoarthritis. Recent studies shown that favorable outcomes are achievable (Table 1) including clinical score improvement throughout 1–7 years of follow-up, and satisfactory quality of cartilage on follow-up MRI and second-look arthroscopy. Clinical improvement after CARTISTEM use was reported to be better than that of both Bone Marrow Aspirate Concentrate (BMAC) and microfracture [52, 53]. According to a study that histologically evaluated cells, the quality of repaired cartilage was similar that of hyaline cartilage [54]. Although more studies are needed, especially those with long-term follow-up periods, CARTISTEM is expected to facilitate the generation of cartilage of improved quality versus existing treatments (Fig. 3).

**Table 1:** Summary of the literature evaluating the use of hUCB-MSC

	Patients (*n*)	Follow-up (years)	Staging	Clinical score	Imaging and second look	Key features of the study
Park et al.	7	7	ICRS4	VAS, IKDC	MRI	Histological evaluation
Na et al.	14	1	ICRS3B	IKDC, KSS, WOMAC	ICRS CRA	Concomitant HTO Better result compared with BMAC (25 cases)
Lim et al.	73	5	ICRS4	VAS, WOMAC, IKDC		Better result compared with microfracture
Song et al.	25	2	ICRS4	VAS, IKDC, WOMAC	ICRS CRA	Concomitant HTO
Song et al.	128	2	ICRS4	VAS, IKDC, WOMAC	MRI (*N* = 34)	Better result for LFC, trochlea compared with MFC
Song et al.	125	3	ICRS4	VAS, WOMAC, IKDC	ICRS CRA	Concomitant HTO

Although ACI or stem cell procedures produce good results [55], single-stage operations that use bioscaffolds or isolated cells from the bone marrow [56, 57] or fat [58] have become popular due to the high cost of ACI and stem cell procedures [59, 60]. These single-stage operations have been actively implemented since the early 2000s. This is because even if microfracture facilitates the generation of fibrocartilage, which has weak mechanical properties, it has advantages such as good short-term clinical results, low economic costs, and simplicity. Therefore, further development of the microfracture technique will be advantageous.

Autologous matrix-induced chondrogenesis (AMIC) is a surgical technique that was developed to enhance arthroscopic microfracture. The procedure is performed as follows: curettage is performed on the cartilage defect area; microfracture is performed on exposed subchondral bone to form a channel through which bone marrow stem cells may move to the damaged area; subsequently, a collagen I/III membrane is attached to the area of damage via suture or using allogenic fibrin glue [61, 62]. Attachment of the collagen membrane after microfracture promotes cartilage formation by sealing bone marrow stem cells within the area of damage. Despite the need for a large incision to attach the collagen membrane to the defect area, Gille et al. reported that 87% of the patients were satisfied with outcomes of the procedure at the 5-year midterm follow-up [63]. A recent study reported the use of gel-type collagen instead of a collagen membrane or allogenic fibrin glue for attachment.

**Fig. 4:**
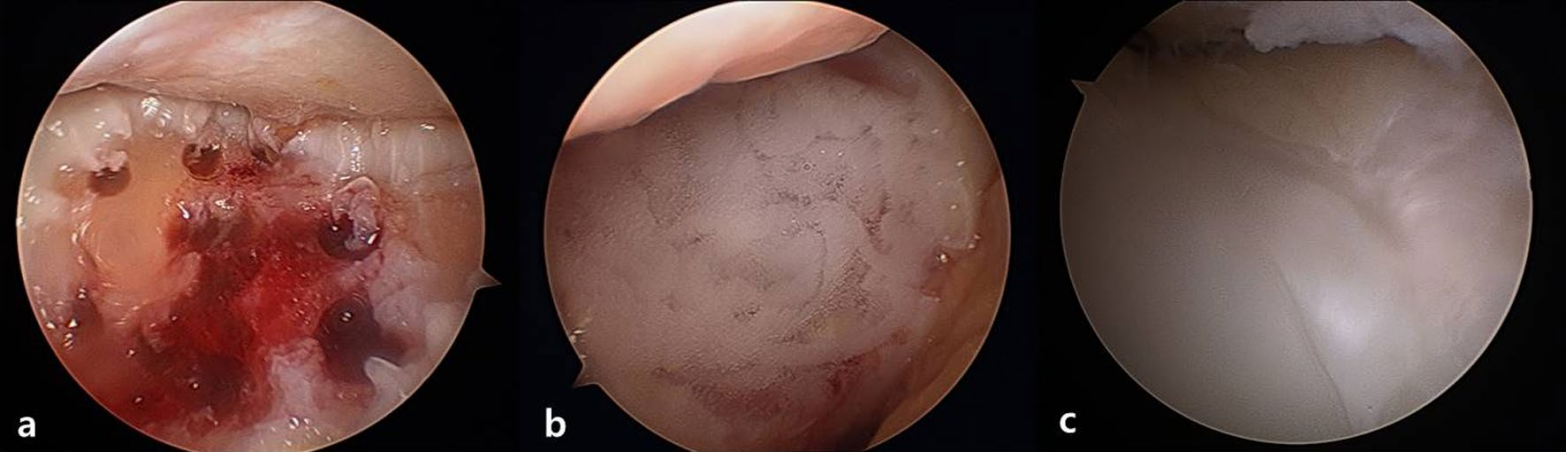
Images of a cartilage defect of trochlear are shown **a** after creation of multiple drilling sites, **b** when CartiRegen® was applied to the defect arthroscopically, and **c** via second-look arthroscopy performed 2-years postsurgery

Kim et al. described the use of autologous collagen-induced chondrogenesis (ACIC) for fixing bone marrow stem cells using a collagen gel after cartilage defect area microfracture. Since the entire procedure is performed in an arthroscopic setting, additional incisions are not required; thus, quick recovery is possible [64–67]. Kim et al. recently reported the results of a 6-year midterm follow-up assessment, which revealed consistent functional score improvement [International Knee Documentation Committee (IKDC), Lysholm, and Knee Injury and Osteoarthritis Outcome Score (KOOS)]. Further, quantitative T2* mapping revealed that repaired and native tissues had the same values, including a mean Magnetic Resonance Observation of Cartilage Repair Tissue (MOCART) score of 78 [standard deviation (SD) = 9.6]. Treatment using manufactured cell products is costly. However, ACIC reduces costs and thus is considered a good treatment option for arthritis (Fig. 4) [64].

## Future

The transition to nonsurgical methods for arthritis treatment is believed to be a critically important feature that characterizes the shift from present to future treatment strategies. Current surgical treatments for damaged cartilage and early osteoarthritis are based on arthroscopic surgical techniques, with or without realignment osteotomy, and involve the application of cells or bioscaffolds to cartilage defects. However, strategies only repair damaged local articular cartilage or a portion of an arthritic joint. Therefore, they are insufficient for treating an entire joint. In addition, current treatments aim to repair joints that are damaged to some extent, including denuded articular cartilage. Therefore, preemptive treatments performed before damage occurs are needed.

A therapeutic agent has recently been developed and commercialized that both slows the progression of arthritis and cures arthritis-related symptoms via intraarticular injection [68, 69]. Thus far, the results of clinical trials have revealed that therapy using this agent is effective. Further, a 3-year follow-up study reported good results. However, it was revealed that 293 cells used were derived from human embryonic kidney cells rather than cartilage cells as previously planned. This revelation put the therapy at risk of being discontinued, however, a phase 3 clinical trial is currently being performed in the USA.

Despite the reported problems, Invossa-K dramatically improved cell agent therapy after its commercialization. Cell agents that have recently been developed and introduced are administered via injection, not surgery. Further, therapeutic effects against arthritis are due to their antiinflammatory, cartilage regeneration promoting activities. Numerous studies have examined the use of distinct cell agents, some of which are in the process of being commercialized [70–72].

Studies examining arthritis initiation have also been conducted. This is considered important because researchers believe that if such a starting point is identified, it may facilitate the prevention of arthritis progression. Therefore, several potential early markers of arthritis have been identified, which may contribute to the initiation of arthritis. Among these are numerous cytokines, chemokines, growth factors, and matrix metalloproteinases. Based on these studies, the inflammatory pathway has been shown to be involved in arthritis progression. For example, it was previously confirmed that *M*1/*M*2 phenotypes of macrophages play an important role in arthritis progression. In addition, studies have assessed outcomes associated with blocking this process and antiinflammatory responses [73–81]. For example, a recent study revealed antiinflammatory and cartilage regenerative effects of the application of stem cells, in which signal transducer and activator of transcription 3 (STAT3) was inhibited. During the progression of arthritis, STAT3 induces the secretion of proinflammatory cytokines. STA21, a small molecule that inhibits the STAT3 pathway, was used to treat mesenchymal stem cells in the treatment of arthritis. STAT3 inhibition enhanced therapeutic effects of mesenchymal stem cells by inhibiting inflammation, which then increased the capacity of cartilage to regenerate [78].

These findings indicate that future arthritis treatments will likely involve nonsurgical therapeutic strategies. For people at high risk of developing arthritis, prophylactic treatments are useful for preventing the onset of arthritis. Many risk factors of joint pain have been identified, which can identify patients at risk of arthritis development who currently are not experiencing pain. The current, well-known risk factors for arthritis include a history of knee joint injury, arthroscopic surgery, or fractures around the knee. Further, athletes, laborers, and individuals with obesity are at increased risk of arthritis development. In the future, patients should take care to inform themselves regarding possible risk factors, and undergo regular screening. Outpatient visits, consultations, and observations are likely good ways for arthritis patients to preserve their joints into old age with minimum effort. Therefore, medical personnel should identify, educate, examine, and treat patients with risk factors of arthritis by forming connections with local community health systems.

## Conclusion

Damaged articular cartilage has a limited ability to heal naturally. Therefore, over the years, many treatments for damage have been developed. In the past, cartilage treatments were passive, both in terms therapeutic effects and methods employed. However, recent developments in areas of cell and tissue engineering have facilitated the introduction of treatments that combine the two areas, with very encouraging results. Stem cell treatment of damaged articular cartilage has proven to be useful via both experiments and clinical trials. To date, the surgical application of therapeutic agents to joints has been the primary treatment option for arthritis management. However, in the future, nonsurgical or prophylactic methods are expected to become mainstream arthritis therapies.

## Data Availability

Not applicable.
